# Age-dependent sex differences in non-stenotic intracranial plaque of embolic stroke of undetermined source

**DOI:** 10.1038/s41598-023-48091-8

**Published:** 2023-11-24

**Authors:** Na Luo, Zi-Yang Shang, Ben-Qiang Yang, George Ntaios, Hui-Sheng Chen

**Affiliations:** 1 Department of Neurology, General Hospital of Northern Theater Command, Shenyang, People’s Republic of China; 2 Department of Radiology, General Hospital of Northern Theater Command, Shenyang, China; 3 Department of Internal Medicine, Faculty of Medicine, School of Health Sciences, University of Thessaly, Larissa, Greece

**Keywords:** Neuroscience, Medical research, Neurology

## Abstract

Age and sex have effect on atherosclerosis. This study aimed to investigate their effect on non-stenotic intracranial atherosclerotic plaque (NIAP) in embolic stroke of undetermined source (ESUS) using high-resolution magnetic resonance imaging (HR-MRI). We retrospectively recruited consecutive ESUS patients who underwent intracranial HR-MRI to assess the plaque characteristics (remodeling index [RI], plaque burden [PB], fibrous cap [FC], discontinuity of plaque surface [DPS], intraplaque hemorrhage [IPH] and complicated plaque [CP]). We divided patients into three groups (< 60 years, 60–74 years, ≥ 75 years). 155 patients with ipsilateral NIAP were found from 243 ESUS patients, with 106 men (68.39%) and 49 women (31.61%). In total population or age group under 60 years, there were no significant differences in plaque characteristics between men and women (all p > 0.05). In age group of 60–74 years, men were associated with higher PB (66.27 ± 9.17% vs 60.91 ± 8.86%, p = 0.017) and RI (1.174 vs 1.156, p = 0.019), higher prevalence of DPS (82.50% vs 60.00%, p = 0.036) and complicated plaque (85.00% vs 63.33%, p = 0.036). For subjects ≥ 75 years old, PB were significantly higher in twomen vs men (68.85 ± 6.14% vs 62.62 ± 7.36%, p = 0.040). In addition, the probability for PB_upper_ (≥ median PB), RI_upper_ (≥ median RI) and vulnerable plaque increased as age increased, and its predictive power for index ESUS was higher in men than women. This study identified age-dependent sex differences in NIAP characteristics of ESUS patients, which will help us clarify their etiology.

## Introduction

In 2014, the Cryptogenic Stroke/ESUS International Working Group proposed the clinical concept of embolic strokes of undetermined source (ESUS)^1^. The criteria and the diagnostic algorithm for ESUS were recently updated^2^.Although there are substantial differences between men and women in terms of stroke occurrence^3^, how the effect of gender on intracranial plaque in patients with ESUS remains unexplored.

It is well known that age and sex have effect on atherosclerosis. Women tend to have higher prevalence of stable plaques compared with men^4^. Men also have more often a plaque with multiple vulnerable plaque components in symptomatic patients with mild-to-moderate carotid stenosis^5^. Plaque prevalence was higher in men than women in most age groups, until the age of 75, when carotid atherosclerosis was more common in women (81.2%) than men (76.5%)^6^. In addition, the effect of age and gender on ischemic stroke risk and pathophysiology is interactive and complex^7^. Since the clinical concept of ESUS was proposed^1^, the nature of these embolic sources is highly heterogeneous^8–11^. Growing evidence presented the importance of non-stenotic atherosclerotic plaques located extracranial^12–14^ or intracranial vessels^15,16^. Recently, a study based computed tomographic angiography (CTA) of the neck suggested that there were gender differences in carotid plaque composition in the ESUS cohort, but no significant difference in the atrial fibrillation (AF) cohort^17^.

In this context, we hypothesized that there may be sex differences in the characteristics of intracranial plaques, which could be affected by age. In the present study, we assess the characteristics of non-stenotic intracranial atherosclerotic plaque (NIAP) in women vs men with ESUS using 3.0 T high-resolution magnetic resonance imaging (MRI) to test the hypothesis.

## Methods

### Study enrollment and information collection

We used the same ESUS cohort, which has been reported in detail in our recent study^15^. We retrospectively recruited patients with acute ischemic stroke in the territory of a unilateral anterior circulation between January 2015 and December 2019. All patients included in the study completed HR-MRI within one week from onset and were eligible for proposed the diagnostic criteria for ESUS. In addition, we further excluded patients with nonstenosing carotid plaque ≥ 3 mm detected by computed tomography angiography (CTA) or carotid ultrasonography, aortic arch atherosclerotic plaque with ulceration or ≥ 4 mm by CTA or transesophageal echocardiogram (TEE), balloon dilatation and stent, previous radiation therapy to head or neck and malignant tumor. The more detailed inclusion/exclusion criteria were reported elsewhere^15^. The study was performed in accordance with the relevant guidelines and regulations, and approved by the Institutional Review Board of General Hospital of Northern Theater Command (IRB: k2019-57), which waived the requirement to obtain informed consent.

According to age, the patients were categorized into three groups: < 60 years group, 60–74 years group, and ≥ 75 years group. The categorization rationale was based on previous studies: (1) a study suggested that plaque prevalence was higher in men than women in most age groups, until the age of 75, when carotid atherosclerosis was more common in women (81.2%) than men (76.5%)^6^; (2) many studies were grouped by the age of 60^18–20^.

### Imaging analysis

**Figure 1:**
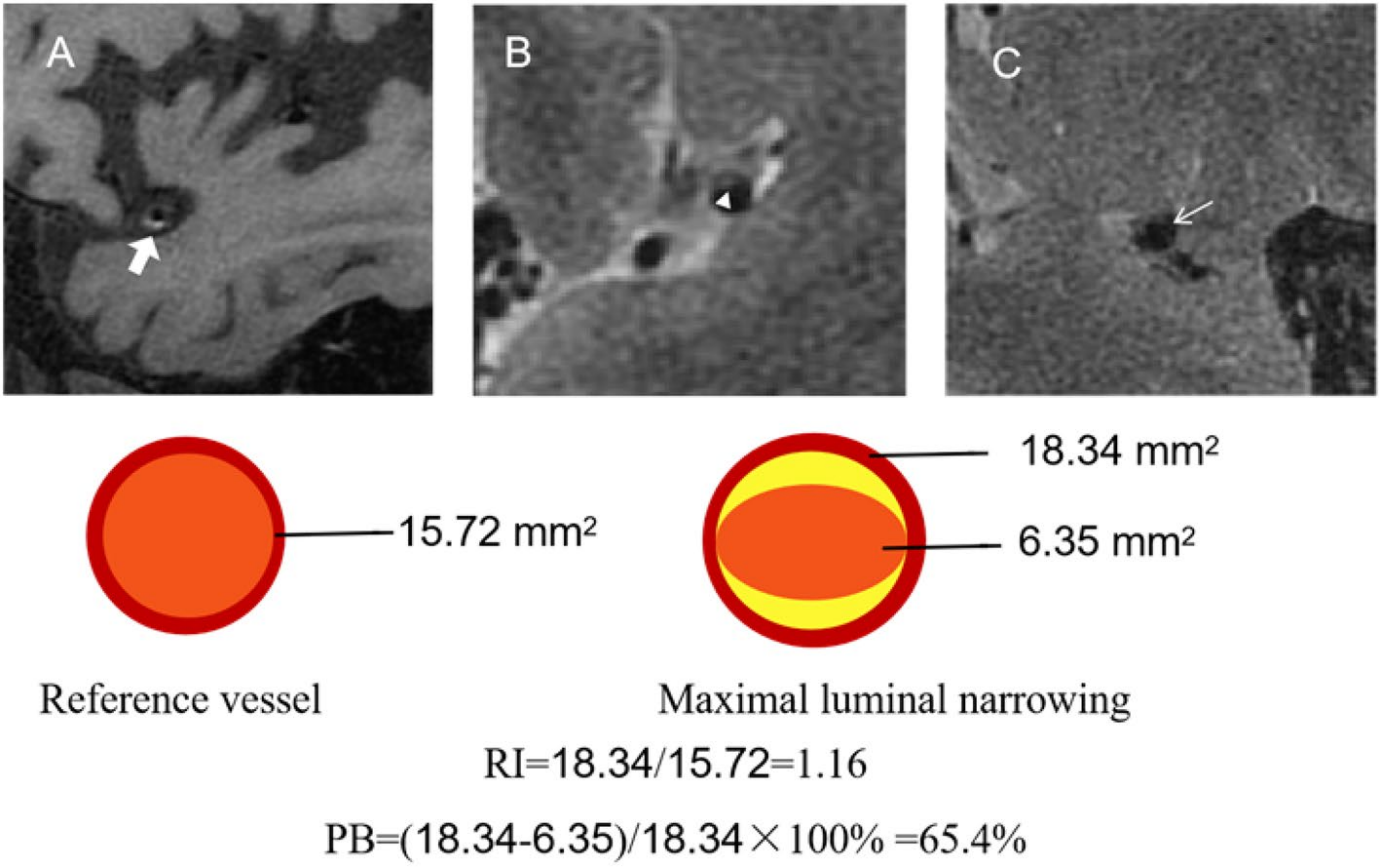
Schematic diagram of multidimensional parameters using 3.0 T high-resolution magnetic resonance imaging. Top: A, the plaque with IPH; B, the plaque with a thick FC; C, the plaque with DPS. Bottom: Schematic diagram of PB and remodeling index RI. IPH, intraplaque hemorrhage; FC, fibrous cap; DPS, discontinuity of plaque surface; PB, plaque burden; RI, remodeling index.

In agreement with our previous study^21^, the culprit plaque was defined as a lesion of ipsilateral proximal vascular territory of infarction with clinical symptoms. The detailed image protocol was reported in our recent study^15,21^. As shown in Fig. 1, multidimensional parameters were evaluated using 3.0 T high-resolution magnetic resonance imaging, including plaque remodeling index (RI), plaque burden (PB), fibrous cap (FC), discontinuity of plaque surface (DPS), intraplaque hemorrhage (IPH) and complicated plaque (CP).

The plaque at the maximal luminal narrowing (MLN) site was selected in the cross-sectional images of vessels, while the reference sites were chosen as the neighboring plaque-free or was calculated as the average of the minimal lesion segment proximal and distal to the MLN site due to vessel tapering. The RI was defined as vessel area at the MLN site divided by reference vessel area, and RI_upper_ was defined as the value ≥ median of remodeling index. The PB was calculated as (vessel area luminal area/vessel area at MLN site) × 100%, and PB_upper_ was calculated as the value ≥ median of plaque burden. The thick FC was defined as a continuous band of T2 high signal adjacent to the lumen. DPS was defined as irregularity of plaque luminal surface, such as FC rupture, ulcer plaque, or formation of overlying mural thrombus. IPH was defined as a bright T1 signal ≥ 150% of T1 signal of adjacent muscle or pons. Complicated plaque (CP) was defined as any or both of DPS and IPH based on the definition of a complicated American Heart Association type VI plaque^22^.

### Statistical analysis

We used Student’s t-test (normally distributed) or the Wilcoxon rank sum test (not normally distributed), as appropriate, for continuous variables, and chi-square test or Fisher’s exact for categorical variables, to compare the difference of baseline clinical, plaque characteristics between men and women among different age groups. To explore the age distribution of plaque characteristics across genders, we performed violin plots and percentage bar charts. To assess probability of vulnerable plaque and facilitate statistical analysis, we transformed PB and RI into binary variables by the median, and then we performed probability curves for plaque characteristics. Univariable and multivariable logistic regression analyses were performed to identify whether vulnerable features of plaques were related to mechanism of the ESUS between men and women. All analyses were performed using SPSS version 22, GraphPad software Inc., Prism Version 8 and a p-value (2-tailed) < 0.05 were considered to indicate statistical significance.

### Ethics approval

This retrospective study was approved by the Institutional Review Board of General Hospital of Northern Theater Command (IRB: k2019-57).

## Results

From the initial cohort of 587 ESUS patients, we excluded patients with bilateral or posterior circulation ESUS (n = 193), carotid plaque of ≥ 3 mm in thickness (n = 91), aortic arch atherosclerosis with ulceration or ≥ 4 mm in thickness (n = 15), paradoxical embolism (n = 27) and poor image quality or incomplete information (n = 18). The final cohort included 243 ESUS patients, of whom 155 (31.6% women) had ipsilateral NIAP and 88 patients had not ipsilateral NIAP.

Supplemental Table 1 summarizes the baseline characteristics in male and female groups. Among ESUS patients with ipsilateral NIAP, compared with men, women were associated with lower prevalence of smoking (14.2% vs 60.3%, p < 0.001) and alcohol drinking (4.0% vs 52.8%, p < 0.001), higher NT-proBNP (126.00 [IQR 63.15–251.2] pg/mL vs 87.17 [IQR 36.04–159.38] pg/mL, p < 0.021), total cholesterol (5.09 ± 1.11 mmol/L vs 4.47 ± 1.18 mmol/L, p = 0.002), HDL (1.06 [0.92–1.29] mmol/L vs 0.94 [0.79–1.08] mmol/L, p < 0.001) and LDL (3.05 ± 0.83 mmol/L vs 2.65 ± 0.81 mmol/L, p < 0.001), but lower creatinine (54.2 [48.87–63.16] umol/L vs 70.55 [62.93–80.90] umol/L, p < 0.001) and homocysteine (10.56 [8.55–13.62] umol/L vs 12.16 [9.96–16.11] umol/L, p < 0.001). We also found similar results in the age group of 60–74 years. In the age group under 60 years, there were differences in smoking, drinking and the level of creatinine between men and women (p < 0.05). For subjects ≥ 75 years old, there was difference in initial NIHSS between men and women (p < 0.05).

**Figure 2:**
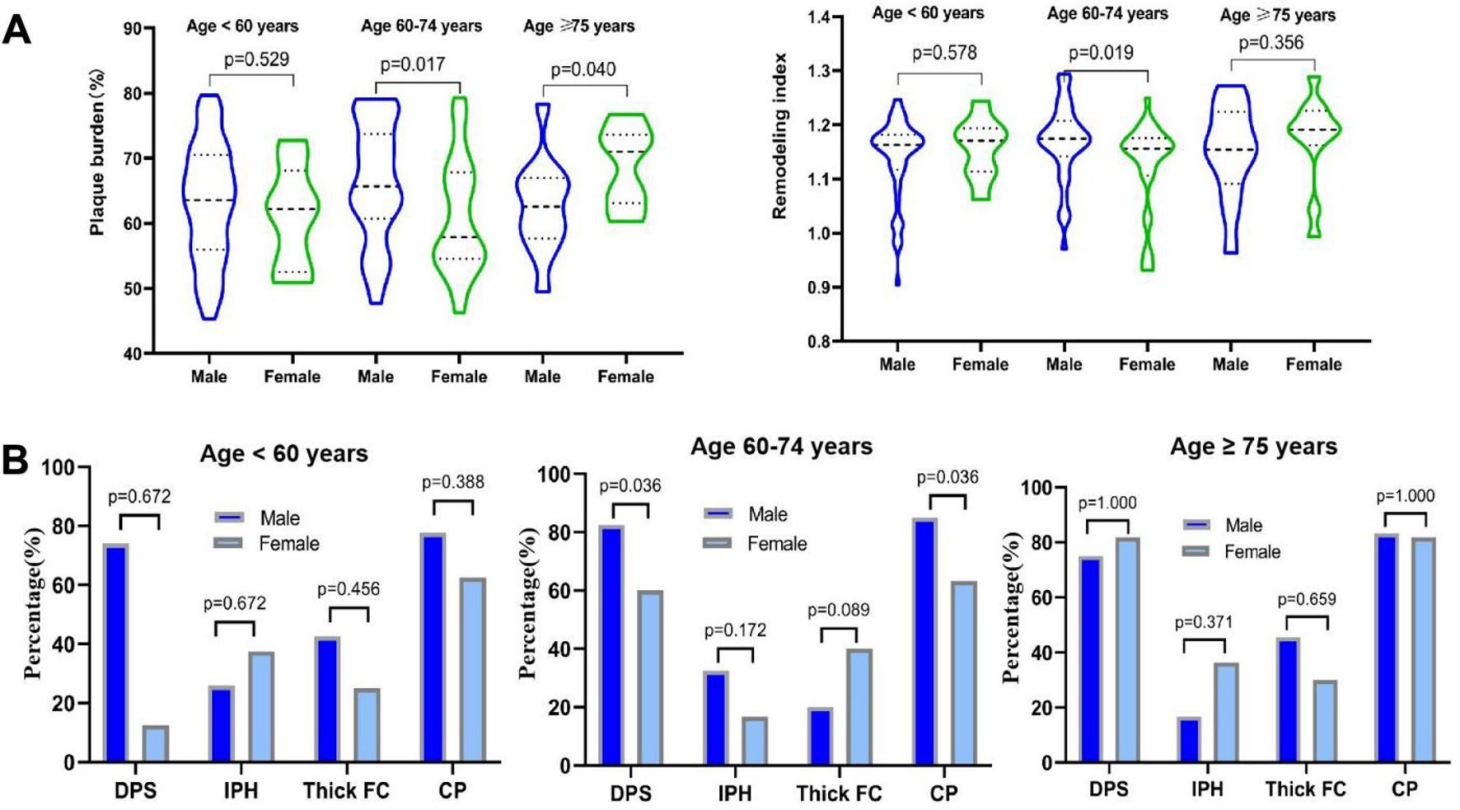
Age-dependent sex differences in ipsilateral NIAP characteristics. Figure A presented age-dependent sex differences in PB and RI; Figure B presented age-dependent probability of DPS, IPH, thick FC or complicated plaque in male vs female ESUS. NIAP, non-stenotic intracranial atherosclerotic plaque; PB, plaque burden; RI, remodeling index; DPS, discontinuity of plaque surface; FC, thick fibrous cap; IPH, intraplaque hemorrhage; CP, complicated plaque.

Table 1 and Fig. 2 summarizes sex differences in ipsilateral NIAP characteristics of ESUS among different age groups. Among all ESUS patients, there was a trend of higher prevalence of complicated plaque in men, which did not reach statistical significance (men vs women: 81.1% vs 67.3%, p = 0.059). In the age group under 60 years, there were no significant differences in plaque characteristics between men and women (all p > 0.05). In the age group of 60–74 years, we found that men were associated with higher PB (66.27 ± 9.17% vs 60.91 ± 8.86%, p = 0.017; Fig. 2A) and RI (1.17 [IQR1.14–1.21] vs 1.16 [1.11–1.18], p = 0.019; Fig. 2A), higher prevalence of DPS (82.50% vs 60.00%, p = 0.036; Fig. 2B) and complicated plaque (85.00% vs 63.33%, p = 0.036; Fig. 2B). For subjects ≥ 75 years old, PB were significantly higher in women vs men (68.85 ± 6.14% vs 62.62 ± 7.36%, p = 0.040; Fig. 2A). There were not sex differences in contralateral NIAP among different age groups (Supplemental Table 2). In addition, we also divided patients into two groups based on the median of age (62 years), and a similar trend was found as the results of three groups (< 60, 60–74, and ≥ 75 years) (Supplemental Table 3).

**Table 1:** Sex differences in ipsilateral NIAP of ESUS among different age groups.

	Total			Age < 60 years			Age 60–74 years			Age ≥ 75 years		
	Male (n = 106)	Female (n = 49)	p	Male (n = 54)	Female (n = 8)	p	Male (n = 40)	Female (n = 30)	p	Male (n = 12)	Female (n = 11)	p
PB, %	64.30 ± 8.98	62.72 ± 8.70	0.304	63.22 ± 9.06	61.08 ± 7.97	0.529	66.27 ± 9.17	60.91 ± 8.86	0.017	62.62 ± 7.36	68.85 ± 6.14	0.040
RI	1.17 (1.13–1.19)	1.16 (1.11–1.19)	0.565	1.16 (1.12–1.18)	1.170 (1.11–1.19)	0.578	1.17 (1.14–1.21)	1.16 (1.11–1.18)	0.019	1.15 (1.09–1.22)	1.19 (1.16–1.23)	0.356
DPS	82 (77.3)	32 (65.3)	0.114	40 (74.07)	1 (12.50)	0.672	33 (82.50)	18 (60.00)	0.036	9 (75.00)	9 (81.82)	1.000
IPH	29 (27.3)	12 (24.4)	0.707	14 (25.93)	3 (37.50)	0.672	13 (32.50)	5 (16.67)	0.172	2 (16.67)	4 (36.36)	0.371
Thick FC	36 (35.0)	17 (35.4)	0.956	23 (42.59)	2 (25.00)	0.456	8 (20.00)	12 (40.00)	0.089	5 (45.45)	3 (30.00)	0.659
CP	86 (81.1)	33 (67.3)	0.059	42 (77.77)	5 (62.50)	0.388	34 (85.00)	19 (63.33)	0.036	10 (83.33)	9 (81.82)	1.000

NIAP,  non-stenotic intracranial atherosclerotic plaque; ESUS,  embolic stroke of undetermined source; PB, plaque burden; RI, remodeling index; DPS, discontinuity of plaque surface; FC, thick fibrous cap; IPH, intraplaque hemorrhage; CP, complicated plaque; values are mean ± SD, median (interquartile range), or n (%).

**Figure 3:**
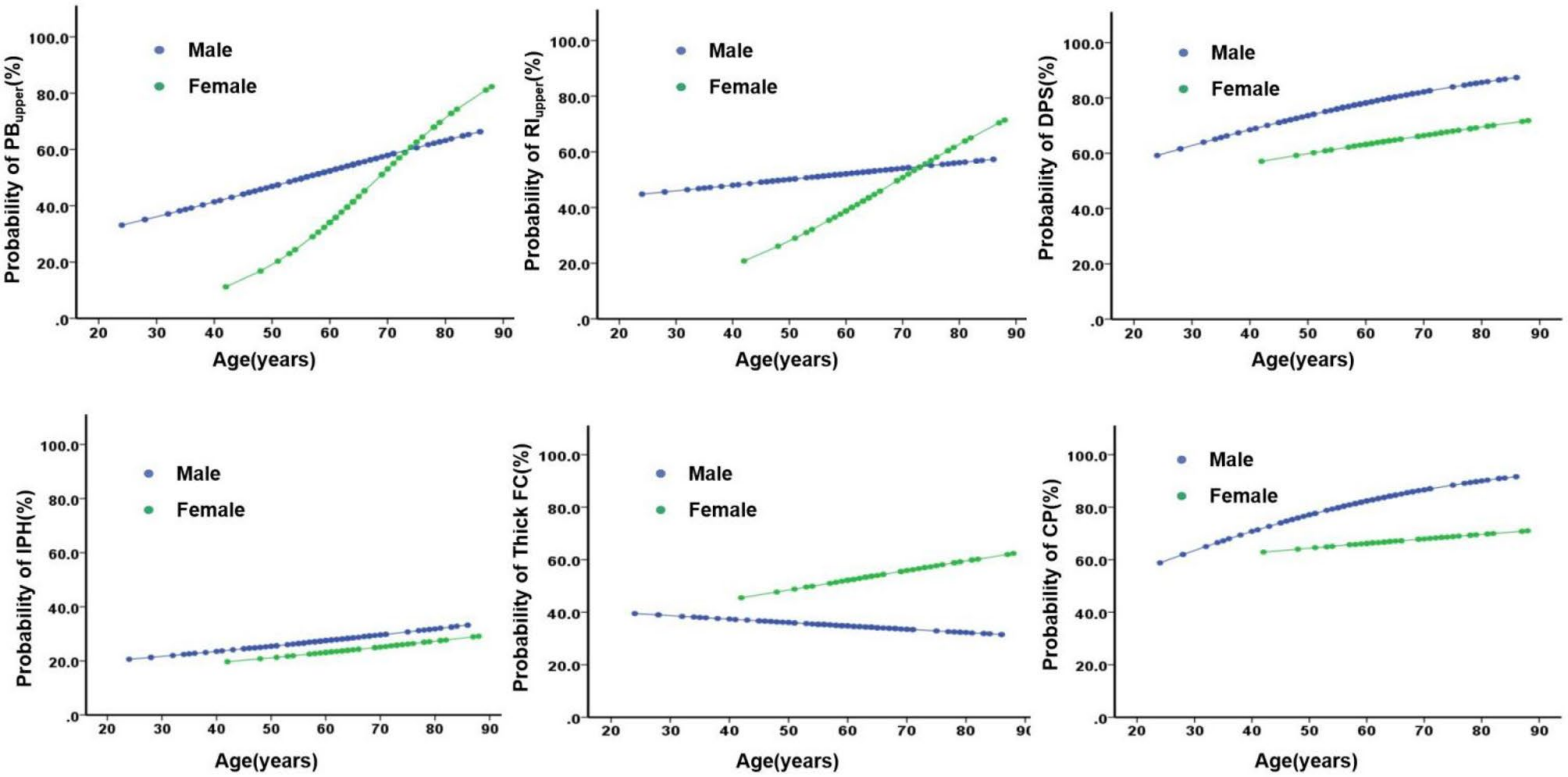
Probability curves for plaque characteristics in male vs female ESUS. PB_upper_, the value ≥ median of plaque burden; RI_upper_, the value ≥ median of remodeling index; DPS, discontinuity of plaque surface; FC, thick fibrous cap; IPH, intraplaque hemorrhage; CP,  complicated plaque.

As shown in Fig. 3, the probability for PB_upper_ and RI_upper_ and vulnerable plaque increased as age increased. Interestingly, the probability of PB_upper_ and RI_upper_ was similar between men and women around age 75. For subjects < 75 years old, the probability of PB_upper_ and RI_upperb_ were higher in men, while the probability of PB_upper_ and RI_upperb_ were higher in women over 75 years old. Furthermore, the probability of DPS, IPH, CP was higher in men regardless of age. In addition, the probability of thick FC was decreased in men as age increased, but increased in women.

**Figure 4:**
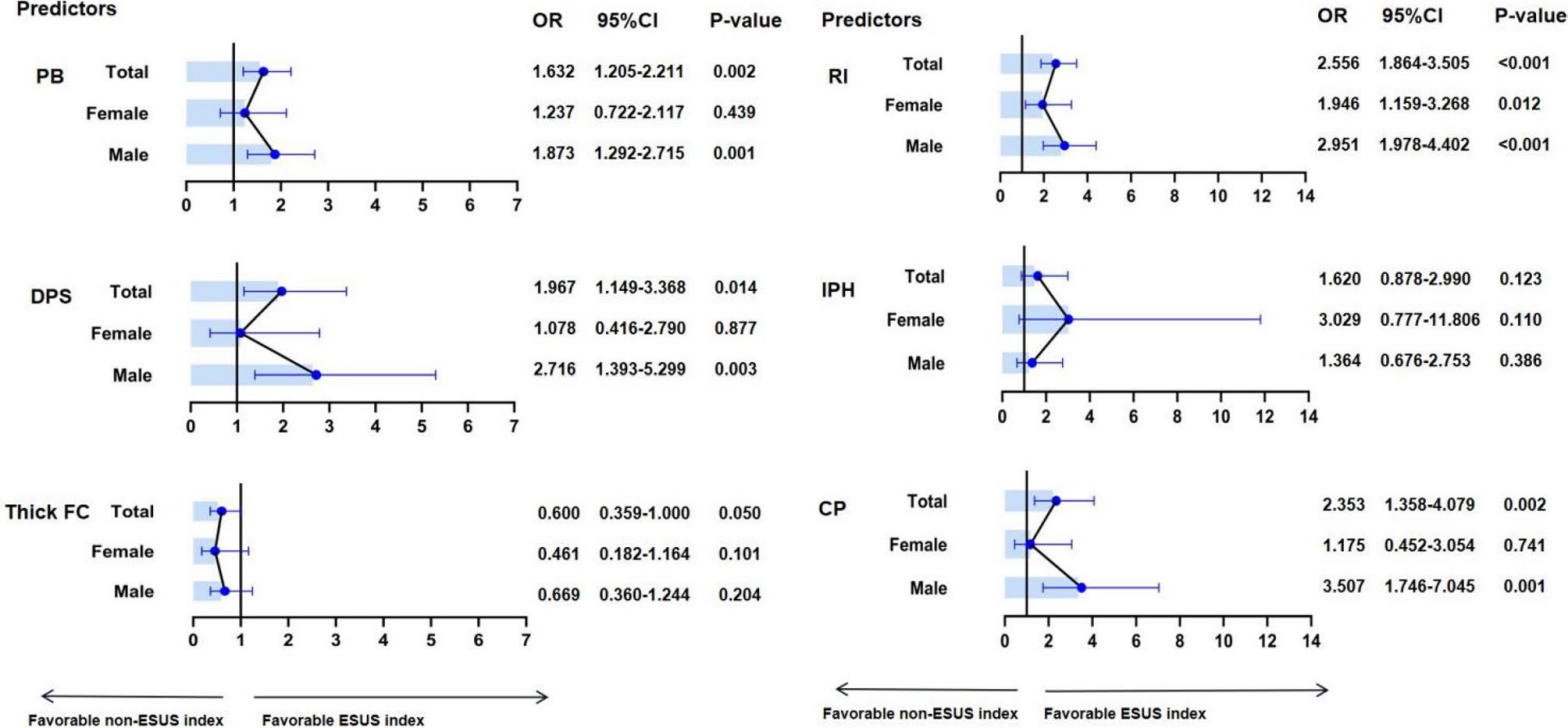
Subgroup analyses by sex stratification for predicting ESUS. All models adjusted for age. PB, plaque burden; RI, remodeling index; DPS, discontinuity of plaque surface; FC, thick fibrous cap; IPH, intraplaque hemorrhage; CP, complicated Plaque.

Table 2 and Fig. 4 summarizes the sex difference of NIAP ipsilateral vs contralateral to ESUS. The univariable logistic regression analyses adjusting for age or excluding more than 75 years old patients showed that PB, RI, DPS and complicated plaque were related with an index ESUS in total patients or in men (all p < 0.05). In women, only RI was related with an index ESUS (p < 0.05), but the association disappeared after excluding more than 75 years old patients (p = 0.120).

**Table 2:** Univariable logistic regression analyses of ipsilateral NIAP for index ESUS, after adjusted for age or age ≤ 75 years.

	Univariable OR (95% CI)	p	Adjust age OR (95% CI)	p	Age ≤ 75 years OR (95% CI)	p
Total						
PB *10	1.593 (1.182–2.149)	0.002	1.632 (1.205–2.211)	0.002	1.449 (1.054–1.992)	0.022
RI*10	2.553 (1.861–3.503)	< 0.001	2.556 (1.864–3.505)	< 0.001	2.387 (1.703–3.346)	< 0.001
DPS	1.884 (1.109–3.200)	0.019	1.967 (1.149–3.368)	0.014	1.920 (1.081–3.411)	0.026
IPH	1.609 (0.872–2.968)	0.128	1.620 (0.878–2.990)	0.123	1.465 (0.760–2.842)	0.255
Thick FC	0.607 (0.365–1.011)	0.055	0.600 (0.359–1.000)	0.050	0.638 (0.365–1.114)	0.114
CP	2.239 (1.304–3.845)	0.003	2.353 (1.358–4.079)	0.002	2.250 (1.254–4.038)	0.007
Male						
PB *10	1.826 (1.266–2.632)	0.001	1.873 (1.292–2.715)	0.001	1.803 (1.232–2.638)	0.002
RI*10	2.962 (1.985–4.421)	< 0.001	2.951 (1.978–4.402)	< 0.001	2.913 (1.905–4.454)	< 0.001
DPS	2.522 (1.317–4.830)	0.005	2.716 (1.393–5.299)	0.003	2.800 (1.406–5.577)	0.003
IPH	1.342 (0.666–2.702)	0.410	1.364 (0.676–2.753)	0.386	1.468 (0.700–3.080)	0.310
Thick FC	0.688 (0.372–1.273)	0.234	0.669 (0.360–1.244)	0.204	0.593 (0.309–1.138)	0.116
CP	3.174 (1.620–6.217)	0.001	3.507 (1.746–7.045)	< 0.001	3.401 (1.673–6.913)	0.001
Female						
PB *10	1.184 (0.702–1.997)	0.527	1.237 (0.722–2.117)	0.439	0.807 (0.436–1.493)	0.495
RI*10	1.897 (1.131–3.179)	0.015	1.946 (1.159–3.268)	0.012	1.541 (0.894–2.657)	0.120
DPS	1.035 (0.404–2.656)	0.942	1.078 (0.416–2.790)	0.877	0.767 (0.251–2.341)	0.641
IPH	3.027 (0.779–11.759)	0.110	3.029 (0.777–11.806)	0.110	1.600 (0.375–6.820)	0.525
Thick FC	0.452 (0.180–1.136)	0.091	0.461 (0.182–1.164)	0.101	0.778 (0.262–2.306)	0.650
CP	1.134 (0.440–2.926)	0.794	1.175 (0.452–3.054)	0.741	0.857 (0.279–2.631)	0.788

NIAP,  non-stenotic intracranial atherosclerotic plaque; ESUS,  embolic stroke of undetermined source; PB, plaque burden; RI, remodeling index; DPS, discontinuity of plaque surface; FC, thick fibrous cap; IPH, intraplaque hemorrhage; CP,  complicated plaque.

**Figure 5:**
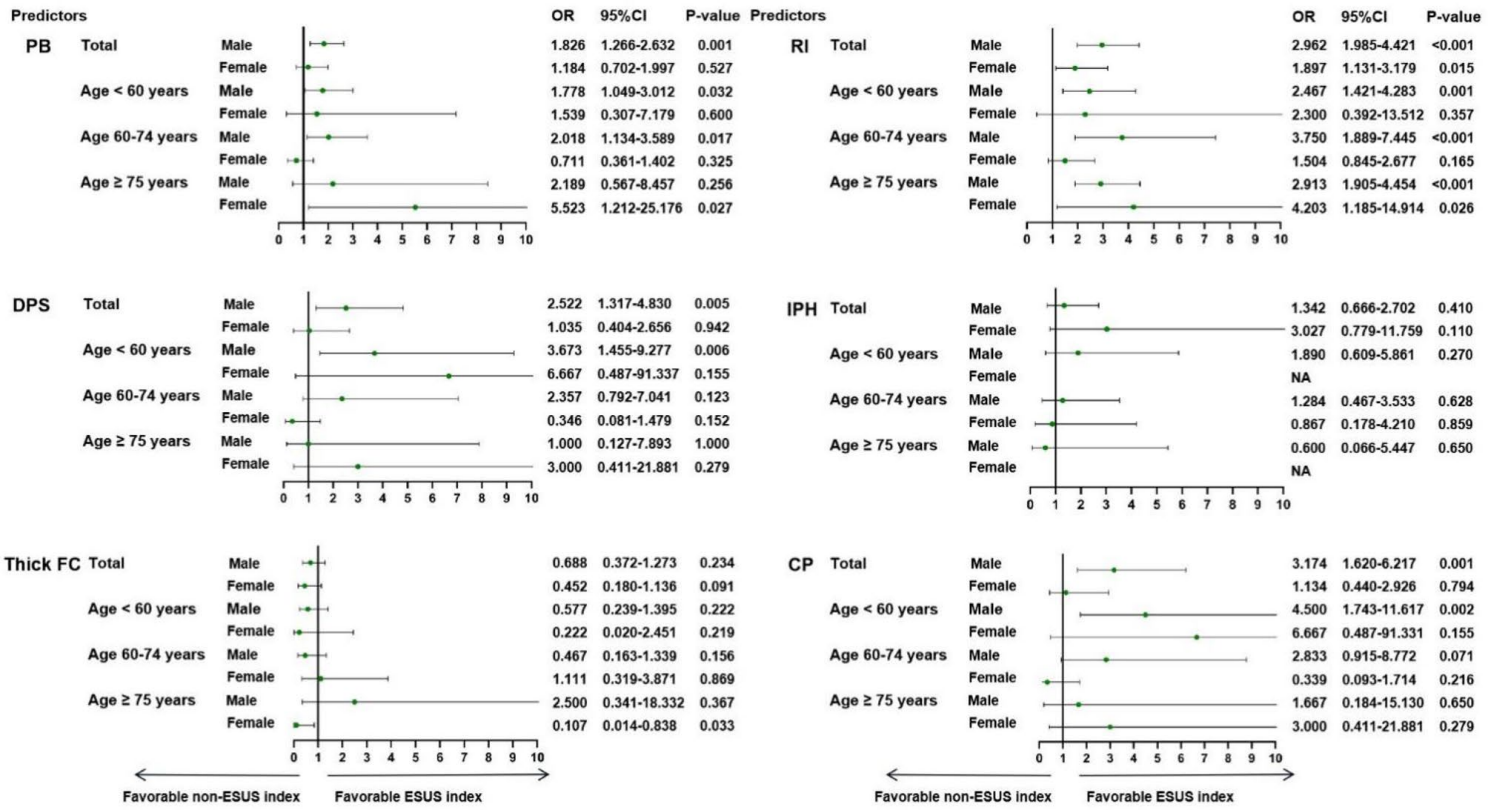
Forest plot of association between plaque characteristics and ESUS index among different age groups. PB,  plaque burden; RI, remodeling index; DPS, discontinuity of plaque surface; FC,  thick fibrous cap; IPH, intraplaque hemorrhage; CP,  complicated Plaque.

**Figure 6:**
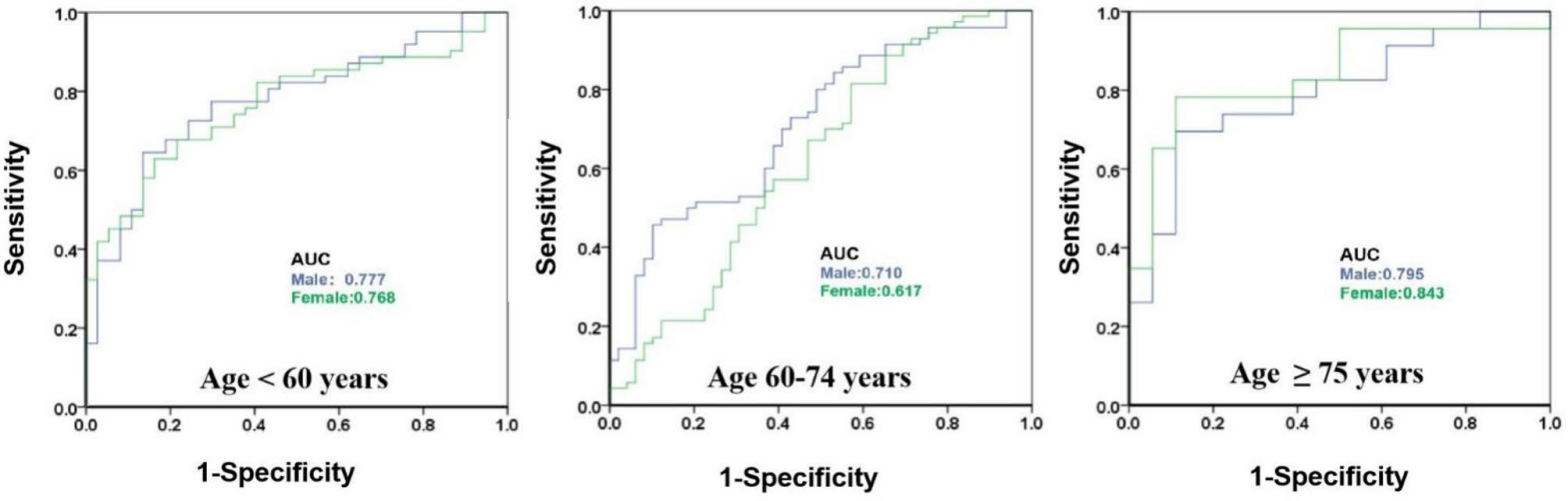
Comparison of ROC analysis of plaque vulnerability among different age groups for predicting ESUS. The model included PB, RI and CP. PB,  plaque burden; RI, remodeling index; CP,  complicated plaque.

Univariable logistic regression analyses were performed to identify the sex difference of ipsilateral vs contralateral NIAP in different age groups (Supplemental Table 4 and Fig. 5). In the age group under 60 years, PB, RI, DPS, and complicated plaque were related with an index ESUS in the overall cohort or in men (p < 0.05), but none of these were related with an index ESUS in women (p > 0.05). In the age group of 60–74 years, RI was related with an index ESUS in total patients (p < 0.05), while PB, RI with an index ESUS in men (p < 0.05), but none of these were related with an index ESUS in women. For subjects ≥ 75 years old, RI was related with an index ESUS in the overall cohort, men or women, and PB were related with an index ESUS in total patients or women. On multivariable logistic regression analysis (Table 3), RI was related with an index ESUS in the overall cohort, men or women, and CP was related with an index ESUS in the overall cohort in the age group under 60 years (p < 0.05). In the age group of 60–74 years, RI was related with an index ESUS in the overall cohort or in men (p < 0.05). For subjects ≥ 75 years old, only RI was related with an index ESUS in the overall cohort (p < 0.05) (Table 3). When combined with PB, RI and complicated plaque for predicting index ESUS (Fig. 6), no significant difference in AUC values was found between men and women (AUC: 0.777 vs o.768) in the age group under 60 years. In the age group of 60–74 years, AUC values were higher in men than women (AUC: 0.710 vs o.617). For subjects ≥ 75 years old, AUC values were higher in women than men, with good predictive power.

**Table 3:** Multivariable logistic regression analyses of ipsilateral NIAP for index ESUS in different age groups.

	Age < 60 years OR (95% CI)	p	Age 60–74 years OR (95% CI)	p	Age ≥ 75 years OR(95% CI)	p
Total						
PB *10	1.262 (0.726–2.193)	0.409	1.036 (0.627–1.710)	0.891	2.756 (0.931–8.158)	0.067
RI*10	1.980 (1.163–3.372)	0.012	2.415 (1.484–3.930)	< 0.001	3.212 (1.301–7.926)	0.011
Complicated Plaque	3.136 (1.217–8.087)	0.018	0.781 (0.304–2.008)	0.608	2.170 (0.353–13.345)	0.403
Male						
PB *10	1.210 (1.789–1.854)	0.382	1.172 (0.577–2.380)	0.661	1.135 (0.239–5.382)	0.873
RI*10	2.554 (1.678–3.888)	< 0.001	3.321 (1.574–7.007)	0.002	4.053 (0.756–21.722)	0.102
Complicated Plaque	1.924 (0.898–4.121)	0.092	1.655 (0.441–6.213)	0.456	0.422 (0.017–10.446)	0.598
Female						
PB *10	1.176 (0.669–2.067)	0.574	0.759 (0.345–1.668)	0.492	159.382 (0.185–137,535.146)	0.141
RI*10	2.300 (1.125–3.148)	0.016	1.765 (0.843–3.696)	0.132	35.876 (0.558–2307.143)	0.092
Complicated Plaque	6.667 (0.324–2.465)	0.155	0.319 (0.064–1.589)	0.163	173.660 (0.105–285,876.507)	0.172

“PB *10”, “RI*10” and “Complicated Plaque” were included in the multivariable analysis. NIAP, non-stenotic intracranial atherosclerotic plaque; PB, plaque burden; RI, remodeling index; DPS, discontinuity of plaque surface; FC, thick fibrous cap; IPH, intraplaque hemorrhage. Values are presented as odds ratio and 95% CIs.

## Discussion

The current study assesses age-dependent sex differences of intracranial plaque characteristics in ESUS, and identifies significant sex difference of vulnerable plaques characteristics in men vs women, as well as an effect of age on the sex differences in plaque characteristics.

We found that there were significant differences in PB, RI, DPS and complicated plaque in women vs men. Men had higher PB and RI, higher prevalence of DPS and complicated plaque. The logistic regression analyses showed that PB, RI, DPS and complicated plaque were related with an index ESUS in the overall cohort or men, but only RI was related with an index ESUS in women. The results were well in agreement with previous studies in which men tend to have a higher prevalence of plaques with vulnerable features (large intraplaque haemorrhage, thin fibrous cap, large lipid core, more inflammatory cells) than women^6,23–27^. The gender difference could be related with secretion of estrogen. It is widely accepted that oestradiol has sex-specific protective effects in women against atherosclerosis, a finding which was also confirmed in experimental animal models^28–31^. Unfortunately, no estrogen-related information was collected in our study. However thick FC and IPH was not found to be a significant predictor for index ESUS, they were related to atherosclerotic plaque as PB, RI, DPS and complicated plaque. We speculate that the difference may be related to the moderate sample size, and the imbalance of sample size among groups.

Furthermore, the sex difference was significantly affected by age in the current study. In the 60–74 year group, the vulnerable characteristics of ipsilateral NIAP were found more frequently in men, however, for patients ≥ 75 years old, the vulnerable characteristics occurred more frequently in women. The results were consistent with previous studies: the Tromsø Study found that carotid plaque prevalence was higher in men than women in most of age groups, but more common in women (81.2%) than men (76.5%) until the age of 75^6^. Also*,* carotid IPH, an unstable plaque component, was shown to have delayed onset in women^32^. Similarly, coronary histopathology study showed that women over 50 were much more likely to have vulnerable plaque than younger, premenopausal women^33^. The protection of oestradiol diminishes with age after menopause, and there was a marked reduction in circulating oestradiol in postmenopausal women^4^.In addition, no significant differences were found between men and women with regard to plaque characteristics in the age group under 60 years, which may be due to the smaller sample size of women vs men (8 vs 54).

In the present study, we found that the prevalence of DPS, IPH, CP was higher in men regardless of age, which further supported the high prevalence of plaque vulnerability in men. However, the probability of PB_upper_ and RI_upper_ was similar between men and women around age 75. Considered together with the high prevalence of vulnerable characteristics in ≥ 75 years old women, these results could suggest the hypothesis that the high prevalence of plaque vulnerability changed from men to women at this time window. Higher plaque burden, positive remodeling and complicated plaques are more likely linked to an embolic stroke, as the respond to the increased plaque burden, and outward expansion of vessel wall will lead to high risk of vulnerability to rupture causing embolic stroke^34,35^. This could provide a possible explanation for the different proportions of gender at different ages of ESUS. Besides, we also found that plaques characteristics exhibited different predictive powers for ESUS index in men vs women, or among different age groups. Collectively, these results suggest that the differential effect of age and gender on the probability of specific mechanisms underlying ESUS could be considered in future studies.

The strength of this study is that it is the first to assess age-dependent sex difference in intracranial plaque characteristics of ESUS, which could facilitate tailored secondary prevention and clinical trial design for ESUS patients. The main limitations are the retrospective nature, the moderate sample size, and the imbalance of sample size of women vs men, which render this finding from the study less powerful. In addition, the gender difference might be related with secretion of estrogen, but no estrogen-related information was collected in our study. Given the high prevalence of intracranial atherosclerotic disease in Chinese populations, the findings may have limited generalizability to other populations.

## Conclusion

This study reported age-related sex differences in the characteristics of intracranial plaques in ESUS patients. The findings could inform secondary prevention strategies and clinical trial design in ESUS.

### Supplementary Information

Supplementary Tables.

## Data Availability

Data are available upon reasonable request. The dataset used and/or analyzed during the current study is available from the corresponding author on reasonable request.
