# Supplementary material for: Age-dependent sex differences in non-stenotic intracranial plaque of embolic stroke of undetermined source
Source: Sci Rep. 2023 Nov 24;13:20652. doi: 10.1038/s41598-023-48091-8 (PMC10673951; doi:10.1038/s41598-023-48091-8)
Supplement: Supplementary file 1 — Supplementary Tables. [file 41598_2023_48091_MOESM1_ESM.docx]

**Supplemental Table 1. Comparison of demographic characteristics and laboratory examination between male and female ESUS**

|  | **Total** | | | **Age** **< 60 years** | | | **Age 60-74 years** | | | **Age ≥ 75 years** | | |
| --- | --- | --- | --- | --- | --- | --- | --- | --- | --- | --- | --- | --- |
|  | **Male**  **(n=106)** | **Female (n=49)** | **P** | **Male**  **(n=54)** | **Female (n=8)** | **P** | **Male**  **(n=40)** | **Female (n=30)** | **P** | **Male**  **(n=12)** | **Female**  **(n=11)** | **P** |
| **Current smoker, n (%)** | 64 (60.3) | 7 (14.2) | <0.001 | 18 (33.33) | 2 (25.00) | 0.019 | 22 (55.00) | 3 (10.00) | <0.001 | 4 (26.66) | 2 (18.18) | 0.640 |
| **Alcohol use, n (%)** | 56 (52.8) | 2 (4.0) | <0.001 | 33 (61.11) | 1 (12.50) | 0.018 | 21 (52.50) | 0 (0.00) | <0.001 | 2 (16.66) | 1 (9.09) | 1.000 |
| **Hypertension, n (%)** | 56 (52.8) | 34 (69.3) | 0.052 | 27 (50.00) | 5 (62.60) | 0.709 | 23 (57.50) | 21 (70.00) | 0.284 | 6 (50.00) | 8 (72.72) | 0.400 |
| **DM, n (%)** | 31 (29.2) | 12 (24.4) | 0.539 | 12 (22.22) | 3 (37.5) | 0.388 | 15 (37.5) | 8 (26.66) | 0.340 | 4 (26.66) | 1 (9.09) | 0.317 |
| **CAD, n (%)** | 12 (11.3) | 10 (20.4) | 0.132 | 6 (11.11) | 1 (12.50) | 1.000 | 4 (10.00) | 6 (20.00) | 0.308 | 1 (8.33) | 3 (27.27) | 0.640 |
| **Prior stroke or TIA, n (%)** | 27 (25.4) | 12 (24.4) | 0.896 | 7 (12.96) | 3 (37.50) | 0.111 | 17 (42.50) | 5 (16.67) | 0.021 | 3 (25.00) | 4 (36.36) | 0.667 |
| **Initial NIHSS** | 3 (1-6) | 3 (1-7) | 0.407 | 2 (1-5) | 1 (1-7) | 0.791 | 4 (2-9) | 4 (1-7) | 0.394 | 1 (0-3) | 3(1-11) | 0.037 |
| **NT-proBNP（pg/ml）** | 87.17 (36.04-159.38) | 126.00 (63.15-251.2) | 0.021 | 54.04  (27.12-102.46) | 56.03  (34.23-126.03) | 0.785 | 106.50  (53.68-186.05) | 116.75  (63.62-207.40) | 0.943 | 184.30  (92.24-486.58) | 264.00  (171.70-481.00) | 0.413 |
| **Serum urea, mmol/L** | 5.21  (4.59-6.46) | 5.25  (4.10-6.37) | 0.495 | 4.98  (4.44-5.96) | 5.79  (4.16-6.21) | 0.614 | 5.49  (4.79-6.75) | 4.64  (3.78-5.79) | 0.005 | 4.94  (4.72-5.70) | 7.26  (5.26-8.63) | 0.079 |
| **Creatinine, umol/L** | 70.55 (62.93-80.90) | 54.2 (48.87-63.16) | <0.001 | 68.41  (61.15-79.38) | 53.60  (49.36-60.50) | 0.002 | 70.55  (63.28-80.93) | 51.20  (47.37-57.43) | <0.001 | 81.32  (71.43-90.60) | 75.80  (56.90-89.32) | 0.487 |
| **Homocysteine, umol/L** | 12.16 (9.96-16.11) | 10.56 (8.55-13.62) | 0.010 | 11.73  (9.17-15.00) | 10.19  (7.74-11.69) | 0.176 | 13.25  (10.17-16.93) | 10.22  (8.13-12.28) | 0.003 | 14.25  (10.82-17.94) | 13.99  (10.23-18.07) | 0.928 |
| **Total cholesterol, mmol/L** | 4.47±1.18 | 5.09±1.11 | 0.002 | 4.61±1.31 | 5.11±1.07 | 0.310 | 4.35±1.04 | 5.08±1.05 | 0.006 | 4.20±0.92 | 5.11±1.36 | 0.074 |
| **Triglyceride, mmol/L** | 1.53  (1.05-2.09) | 1.54  (1.03-1.96) | 0.786 | 1.69  (1.22-2.51) | 2.21  (1.09-2.82) | 0.495 | 1.43  (1.04-2.11) | 1.33  (1.01-1.92) | 0.776 | 0.99  (0.84-1.44) | 1.54  (1.13-1.77) | 0.051 |
| **HDL, mmol/L** | 0.94  (0.79-1.08) | 1.06  (0.92-1.29) | <0.001 | 0.96  (0.83-1.07) | 1.03  (0.96-1.27) | 0.072 | 0.92  (0.76-1.07) | 1.06  (0.92-1.29) | 0.006 | 0.91  (0.79-1.15) | 1.08  (0.89-1.42) | 0.235 |
| **LDL, mmol/L** | 2.65±0.81 | 3.05±0.83 | 0.005 | 2.74±0.89 | 3.05±0.94 | 0.356 | 2.58±0.73 | 3.03±0.79 | 0.015 | 2.48±0.72 | 3.09±0.95 | 0.092 |
| **Fibrinogen, g/L** | 3.15  (2.71-3.82) | 3.21 (2.83-3.73) | 0.855 | 3.11  (2.53-3.75) | 3.15  (2.79-3.58) | 0.916 | 3.19  (2.81-3.79) | 3.20  (2.83-3.87) | 0.887 | 3.73  (2.91-4.56) | 3.30  (2.77-3.52) | 0.413 |
| **Lipoprotein A, mg/L** | 126.40  (74.83-269.30) | 172.1  (97.9 354.0) | 0.052 | 129.45  (71.73-281.90) | 155.40  (83.35-591.23) | 0.324 | 116.50  (70.43-176.20) | 166.85  (100.30-322.18) | 0.037 | 194.40  (92.53-439.65) | 198.00  (98.80-434.90) | 0.880 |

ESUS= embolic stroke of undetermined source; DM= Diabetes mellitus; CAD= coronary artery disease; TIA= transient ischemic attack; NT-proBNP=N-terminal probrain natriuretic peptide; HDL= High density lipoprotein; LDL= Low density lipoprotein; Values are mean±SD, median (interquartile range), or n/N (%); missing values 9/243), and the missing values are replaced by the median.

**Supplemental Table 2. Sex differences in contralateral NIAP of ESUS in different age groups**

|  | **Age < 60 years** | | | **Age 60-74 years** | | | **Age ≥ 75 years** | | |
| --- | --- | --- | --- | --- | --- | --- | --- | --- | --- |
|  | **Male**  **(n=32)** | **Female**  **(n=5)** | **P** | **Male**  **(n=33)** | **Female**  **(n=16)** | **P** | **Male**  **(n=8)** | **Female**  **(n=10)** | **P** |
| **PB, %** | 58.87±8.23 | 58.94±6.99 | 0.985 | 61.176±7.69 | 63.69±9.50 | 0.326 | 58.61±7.84 | 59.12±9.02 | 0.902 |
| **RI** | 1.094  (1.006-1.157) | 1.154  (1.049-1.172) | 0.267 | 1.057  (0.998-1.163) | 1.143 (0.999-1.163) | 0.616 | 1.034 (0.981-1.144) | 1.052 (1.011-1.153) | 0.534 |
| **DPS** | 14 (43.75) | 1 (20.00) | 0.629 | 22 (66.67) | 13 (81.25) | 0.336 | 6 (75.00) | 6 (60.00) | 0.628 |
| **IPH** | 5 (15.63) | 0 (00.00) | 1.000 | 11 (39.29) | 3 (18.75) | 0.726 | 2 (25.00) | 0 (00.00) | 0.183 |
| **Thick FC** | 18 (56.25) | 3 (60.00) | 1.000 | 6 (21.43) | 6 (37.50) | 0.938 | 2 (25.00) | 8 (80.00) | 0.054 |
| **Complicated plaque** | 14 (43.75) | 1 (20.00) | 0.629 | 22 (66.67) | 13 (81.25) | 0.336 | 6 (75.00) | 6 (60.00) | 0.638 |

ESUS= embolic stroke of undetermined source; NIAP = non-stenotic intracranial atherosclerotic plaque; PB= plaque burden; RI= remodeling index; DPS= discontinuity of plaque surface; FC= thick fibrous cap; IPH= intraplaque hemorrhage; Values are mean±SD, median (interquartile range), or n (%)

**Supplemental Table 3. Sex differences in ipsilateral NIAP of ESUS among different age groups**

|  | **Age < 62 years** | | | **Age ≥ 62 years** | | |
| --- | --- | --- | --- | --- | --- | --- |
|  | **Male (n=61)** | **Female (n=16)** | **P** | **Male (n=45)** | **Female (n=33)** | **P** |
| **PB, %** | 63.43±9.69 | 59.51±6.31 | 0.059 | 65.49 ±7.88 | 64.28±9.34 | 0.535 |
| **RI** | 1.16 (1.12-1.18) | 1.16 (1.08-1.18) | 0.547 | 1.18 (1.14-1.22) | 1.17 (1.13-1.19) | 0.317 |
| **DPS** | 45 (73.77) | 11 (68.75) | 0.756 | 37 (82.22) | 21 (63.64) | 0.063 |
| **IPH** | 17 (27.87) | 5 (31.25) | 0.765 | 12 (26.67) | 7 (21.21) | 0.579 |
| **Thick FC** | 26 (42.62) | 4 (25.00) | 0.256 | 10 (23.81) | 13 (40.63) | 0.122 |
| **CP** | 47 (77.05) | 12 (75.00) | 1.000 | 39 (86.67) | 21 (63.64) | 0.017 |

NIAP = non-stenotic intracranial atherosclerotic plaque; ESUS= embolic stroke of undetermined source; PB= plaque burden; RI= remodeling index; DPS= discontinuity of plaque surface; FC= thick fibrous cap; IPH= intraplaque hemorrhage; CP= Complicated plaque; Values are mean±SD, median (interquartile range), or n (%).

**Supplemental Table 4. Univariable logistic regression analyses of ipsilateral NIAP for index ESUS in different age groups**

|  | **Age < 60 years**  **OR (95% CI)** | **P** | **Age 60-74 years**  **OR (95% CI)** | **P** | **Age > 75 years**  **OR（95% CI）** | **P** |
| --- | --- | --- | --- | --- | --- | --- |
| **Total** | | | | | | |
| **PB *10** | 1.754  (1.063-2.891) | 0.028 | 1.287  (0.848-1.955) | 0.236 | 3.182  (1.218-8.308) | 0.018 |
| **RI*10** | 2.410  (1.432-4.055) | 0.001 | 2.374  (1.523-3.702) | <0.001 | 3.653  (1.552-8.599) | 0.003 |
| **DPS** | 3.882  (1.641-9.188) | 0.002 | 1.074  (0.476-2.422) | 0.864 | 1.800  (0.447-7.253) | 0.408 |
| **IPH** | 2.418  (0.809-7.229) | 0.114 | 1.067  (0.459-2.481) | 0.880 | 2.824  (0.496-16.084) | 0.242 |
| **Thick FC** | 0.515  (0.226-1.175) | 0.115 | 0.718  (0.329-1.566) | 0.405 | 0.492  (0.137-1.772) | 0.278 |
| **Complicated**  **Plaque** | 4.596  (1.913-11.043) | 0.001 | 1.247  (0.546-2.849) | 0.600 | 2.375  (0.553-10.196) | 0.245 |
| **Male** | | | | | | |
| **PB *10** | 1.778  (1.049-3.012) | 0.032 | 2.018  (1.134-3.589) | 0.017 | 2.189  (0.567-8.457) | 0.256 |
| **RI*10** | 2.467  (1.421-4.283) | 0.001 | 3.750  (1.889-7.445) | <0.001 | 2.913  (1.905-4.454) | <0.001 |
| **DPS** | 3.673  (1.455-9.277) | 0.006 | 2.357  (0.792-7.041) | 0.123 | 1.000  (0.127-7.893) | 1.000 |
| **IPH** | 1.890  (0.609-5.861) | 0.270 | 1.284  (0.467-3.533) | 0.628 | 0.600  (0.066-5.447) | 0.650 |
| **Thick FC** | 0.577  (0.239-1.395) | 0.222 | 0.467  (0.163-1.339) | 0.156 | 2.500  (0.341-18.332) | 0.367 |
| **Complicated**  **Plaque** | 4.500  (1.743-11.617) | 0.002 | 2.833  (0.915-8.772) | 0.071 | 1.667  (0.184-15.130) | 0.650 |
| **Female** | | | | | | |
| **PB *10** | 1.539  (0.307-7.179) | 0.600 | 0.711  (0.361-1.402) | 0.325 | 5.523  (1.212-25.176) | 0.027 |
| **RI*10** | 2.300  (0.392-13.512) | 0.357 | 1.504  (0.845-2.677) | 0.165 | 4.203  (1.185-14.914） | 0.026 |
| **DPS** | 6.667  (0.487-91.337) | 0.155 | 0.346  (0.081-1.479) | 0.152 | 3.000  (0.411-21.881) | 0.279 |
| **IPH** | NA | NA | 0.867  (0.178-4.210) | 0.859 | NA | NA |
| **Thick FC** | 0.222  (0.020-2.451) | 0.219 | 1.111  (0.319-3.871) | 0.869 | 0.107  (0.014-0.838) | 0.033 |
| **Complicated**  **Plaque** | 6.667  (0.487-91.331) | 0.155 | 0.339  (0.093-1.714) | 0.216 | 3.000  (0.411-21.881) | 0.279 |

NIAP = non-stenotic intracranial atherosclerotic plaque; ESUS= embolic stroke of undetermined source; PB= plaque burden; RI= remodeling index; DPS= discontinuity of plaque surface; FC= thick fibrous cap; IPH= intraplaque hemorrhage. Values are presented as odds ratio and 95% CIs.
